# Anemia and adverse outcomes in pregnancy: subgroup analysis of the CLIP cluster-randomized trial in India

**DOI:** 10.1186/s12884-022-04714-y

**Published:** 2022-05-13

**Authors:** Jeffrey N. Bone, Mrutyunjaya Bellad, Shivaprasad Goudar, Ashalata Mallapur, Umesh Charantimath, Umesh Ramadurg, Geetanjali Katageri, Maria Lesperance, Mai-Lei Woo Kinshella, Raiya Suleman, Marianne Vidler, Sumedha Sharma, Richard Derman, Laura A. Magee, Peter von Dadelszen, the CLIP working group, Shashidhar G. Bannale, Keval S. Chougala, Vaibhav B. Dhamanekar, Anjali M. Joshi, Namdev A. Kamble, Gudadayya S. Kengapur, Uday S. Kudachi, Sphoorthi S. Mastiholi, Geetanjali I Mungarwadi, Esperança Sevene, Khátia Munguambe, Charfudin Sacoor, Eusébio Macete, Helena Boene, Felizarda Amose, Orvalho Augusto, Cassimo Bique, Ana Ilda Biz, Rogério Chiaú, Silvestre Cutana, Paulo Filimone, Emília Gonçálves, Marta Macamo, Salésio Macuacua, Sónia Maculuve, Ernesto Mandlate, Analisa Matavele, Sibone Mocumbi, Dulce Mulungo, Zefanias Nhamirre, Ariel Nhancolo, Cláudio Nkumbula, Vivalde Nobela, Rosa Pires, Corsino Tchavana, Anifa Vala, Faustino Vilanculo, Rahat N. Qureshi, Sana Sheikh, Zahra Hoodbhoy, Imran Ahmed, Amjad Hussain, Javed Memon, Farrukh Raza, Olalekan O. Adetoro, John O. Sotunsa, Sharla K. Drebit, Chirag Kariya, Mansun Lui, Diane Sawchuck, Ugochi V. Ukah, Mai-Lei Woo Kinshella, Shafik Dharamsi, Guy A. Dumont, Tabassum Firoz, Ana Pilar Betrán, Susheela M. Engelbrecht, Veronique Filippi, William A. Grobman, Marian Knight, Ana Langer, Simon A. Lewin, Gwyneth Lewis, Craig Mitton, Nadine Schuurman, James G. Thornton, France Donnay, Romano N. Byaruhanga, Brian Darlow, Eileen Hutton, Mario Merialdi, Lehana Thabane, Kelly Pickerill, Avinash Kavi, Chandrashekhar Karadiguddi, Sangamesh Rakaraddi, Amit Revankar

**Affiliations:** 1 Department of Obstetrics and Gynaecology, University of British Columbia, Vancouver, Canada; 2 British Columbia Children’s Hospital Research Institute, Vancouver, Canada; 3 KLE Academy of Higher Education and Research’s J N Medical College, Belagavi, Karnataka India; 4 S Nijalingappa Medical College and Hanagal Shree Kumareshwar Hospital and Research Center, Bagalkote, India; 5 Richmond Division of Family Practice, Vancouver Coastal Health, Vancouver, Canada; 6 Faculty of Medicine, University of British Columba, Vancouver, Canada; 7 Global Affairs, Thomas Jefferson University, Philadelphia, USA; 8 Department of Women and Children’s Health, School of Life Course Sciences, Faculty of Life Science and Medicine, Kings College London, London, UK

**Keywords:** Anemia in pregnancy, Hypertension, Global health

## Abstract

**Background:**

Iron-deficiency anemia is a known risk factor for several adverse perinatal outcomes, but data on its impact on specific maternal morbidities is less robust. Further, information on associations between anemia in early pregnancy and subsequent outcomes are understudied.

**Methods:**

The study population was derived from the Community Level Interventions for Pre-eclampsia (CLIP) trial in Karnataka State, India (NCT01911494). Included were women who were enrolled in either trial arm, delivered by trial end date, and had a baseline measure of hemoglobin (Hb). Anemia was classified by WHO standards into four groups: none (Hb ≥ 11 g/dL), mild (10.0 g/dL ≤ Hb < 11.0 g/dL), moderate (7.0 g/dL ≤ Hb < 10.0 g/dL) and severe (Hb < 7.0 g/dL). Targeted maximum likelihood estimation was used to estimate confounder-adjusted associations between anemia and a composite (and its components) of adverse maternal outcomes, including pregnancy hypertension. E-values were calculated to assess robustness to unmeasured confounding.

**Results:**

Of 11,370 women included, 10,066 (88.5%) had anemia, that was mild (3690, 32.5%), moderate (6023, 53.0%), or severe (68, 0.6%). Almost all women (> 99%) reported taking iron supplements during pregnancy. Blood transfusions was more often administered to those with anemia that was mild (risk ratio [RR] 2.16, 95% confidence interval [CI] 1.31–3.56), moderate (RR 2.37, 95% CI 1.56–3.59), and severe (RR 5.70, 95% CI 3.00–10.85). No significant association was evident between anemia severity and haemorrhage (antepartum or postpartum) or sepsis, but there was a U-shaped association between anemia severity and pregnancy hypertension and pre-eclampsia specifically, with the lowest risk seen among those with mild or moderate anemia.

**Conclusion:**

In Karnataka State, India, current management strategies for mild-moderate anemia in early pregnancy are associated with similar rates of adverse maternal or perinatal outcomes, and a lower risk of pregnancy hypertension and preeclampsia, compared with no anemia in early pregnancy. Future research should focus on risk mitigation for women with severe anemia, and the potential effect of iron supplementation for women with normal Hb in early pregnancy.

**Supplementary Information:**

The online version contains supplementary material available at 10.1186/s12884-022-04714-y.

## Introduction

Anemia in pregnancy is a global health concern with the burden falling on low- and middle-income countries (LMIC) in Africa and Southeast Asia, with rates more than twice as high as in high-income settings [1–4]. Iron deficiency is the most common cause of anemia in pregnancy; other causes include deficiency of B12 or folic acid, thalassemias, intrinsic red blood cell disorders, bacterial and parasitic infections [1]. In India specifically, although the prevalence of anemia during pregnancy has been decreasing as of 2018, rates remain high [2, 5].

Maternal anemia has well established connections with adverse neonatal outcomes such as low birth weight, small for gestational age and premature delivery [6–9]. Further, recent evidence from diverse LMIC settings found maternal deaths to be nearly twice as high in individuals with severe anemia [10]. There are also published data linking anemia with pre-eclampsia, puerperal sepsis, ante-partum hemorrhage (APH), and post-partum hemorrhage [11–15]. These studies have well-documented limitations, and their remains a need for high-quality prospective data to further understanding of the associations between severity of anemia and adverse maternal outcomes [10, 16]. Furthermore, although iron supplementation has been shown to have protective effects of maternal anemia (at delivery) and low birthweight, there is little evidence for its impact on other adverse pregnancy outcomes [17–19].

Using data from over 10,000 pregnancies in the Community Level Interventions for Pre-Eclampsia (CLIP) trial in Karnataka State, India [20] these analyses measure the prevalence of anemia and iron supplementation and assess the impact of anemia severity in early pregnancy on adverse maternal outcomes including need for blood transfusion, ante-partum hemorrhage, and hypertension.

## Methods

This was an unplanned secondary analysis using data from the CLIP India trial [20] with the goal of estimating the effect of early pregnancy anemia on maternal and perinatal outcomes in a population with high rates of iron supplementation.

### CLIP India trial

The CLIP India trial was a prospective, cluster randomized control trial that took place in 12 clusters in Belagavi and Bagalkote districts, rural Karnataka. The units of randomization were primary health care centers and were chosen by the site teams based on a variety of feasibility and logistical considerations. Four clusters (two per arm) were included in a pilot phase from February 1^st^, 2014 to October 31^st^, 2014. These four clusters were then joined by the additional eight for a two-year definitive phase from November 1^st^, 2014 to October 31^st^, 2016.

Participants were married pregnant women (ages 15–49) who provided written informed consent for data collection. In both the intervention and control clusters, data was obtained via household and facility-based surveys. These surveys were conducted by community health care workers and research staff to collect socio-demographic characteristics, care seeking behaviours, maternal outcomes and neonatal outcomes. Data was collected at three time points, (i) as soon as possible after enrolment after pregnancy confirmation (focus on obstetric and medical and previous pregnancy history), (ii) as soon as possible after delivery (focus on care seeking, delivery information and pregnancy outcome for current pregnancy), and (iii) within 42 days postpartum (confirmation and update of pregnancy outcome). Data collection was done through the Maternal and Newborn Health (MNH) registry system [21].

Women in the intervention clusters received additional ‘CLIP visits’ during both the antenatal and postnatal periods. These visits were conducted by community health care workers and were guided by the PIERS on the Move (POM) mobile health technology risk stratification tool [22]. Depending on blood pressure measure and other risk factors, POM provided recommendations around need to 1) continue routine care, 2) seek non-emergent care (within 24 h) or 3) seek emergency care (immediately). Further, in cases with high blood pressure (> 160/110) or preeclampsia, oral methyldopa (750 mg) or intramuscular magnesium sulphate (10 g) was administered, respectively. The intervention clusters also had community engagement sessions for women and other community members to promote knowledge about risks and symptoms of pregnancy hypertension.

### Inclusion and exclusion criteria

The inclusion criteria for the analyses were women who had delivered by the end of the trial (October 31^st^, 2016) with a hemoglobin measurement at trial enrolment.

### Exposure, outcomes and confounders

Hemoglobin level was collected at trial enrolment, which for the majority of women was during the first trimester. Hemoglobin was assessed using Sahli’s method. Maternal anemia was defined as hemoglobin (Hb) < 11 g/dl and severity was further subdivided based on World Health Organization (WHO) standards into: mild (10.0 g/dL ≤ Hb < 11.0 g/dL), moderate (7.0 g/dL ≤ Hb < 10.0 g/dL) and severe (Hb < 7.0 g/dL).

We analyzed several maternal outcomes. First, we looked at those that have been commonly associated with anemia, including blood transfusion, antepartum hemorrhage, maternal sepsis, and postpartum hemorrhage. Outcomes were self-reported and clinically adjudicated by a group of (non-treating) physicians for accuracy as part of the CLIP trial [20]. The only exception was postpartum hemorrhage, which was collected within the MNH registry, which is based on treating physician diagnosis. Second, we assessed pregnancy hypertension (defined as systolic blood pressure ≥ 140 and/or diastolic blood pressure ≥ 90). Blood pressure data were only available on women in the intervention arm, so analyses of hypertension were restricted to this group. Data were collected during POM visits using standardised methods and a semi-automated, pregnancy-validated digital device (Microlife BP 3AS1-2) [23]. In addition to defining hypertension based strictly from POM measurements, we also used a previously published definition (for this data) to combine information from POM and hypertension reported in trial surveillance data sources to assess an expanded version of hypertension, as well as pre-eclampsia [24]. Pre-eclampsia was defined as gestational hypertension with proteinuria or 1 or more relevant end-organ complications [24].

Secondary outcomes included severe perinatal outcomes including perinatal death, stillbirth, early and late neonatal death, neonatal morbidity, and a composite of these outcomes.

Possible confounders were identified based on expert knowledge and possible relationship between anemia and adverse outcomes [25] (Figure S1). These include trial arm, cluster, maternal age, nulliparity, body-mass-index (measured at enrolment), maternal and husband basic education (as measure of socioeconomic status), gestational age at booking (as a measure of access to care), religion (as a possible proxy for vegetarian diet), and twin pregnancy.

Information on iron supplementation was only collected postpartum, and timing of initiation was not ascertained. We did not adjust for iron supplementation as it occurred after exposure and therefore may be on the causal pathway between exposure and outcome. Furthermore, almost all women (> 99%) who delivered reported taking supplementation, but timing and adherence were not available.

### Statistical analysis

The rates of women with no, mild, moderate, and severe anemia were estimated. Demographic and clinical outcomes were stratified across anemic groups and summarized as counts and percentages for categorical variables and medians and IQR for continuous variables. For each anemic group, iron supplementation rates were compared between women with miscarriages and MTP and those whose pregnancies ended in live or stillbirth. Women with medically terminated pregnancies or miscarriages were excluded from analysis of maternal and perinatal outcomes.

We used Targeted Maximum Likelihood Estimation (TMLE) to estimate risk ratios and risk differences between non-anemic women and women with mild and moderate anemia on our primary outcomes of interest [26]. TMLE has the advantage over traditional regression (e.g., logistic) models in that it combines a propensity score model for the exposure (anemia) and a model for the outcome and if either of these models is correctly specified the estimate of association is unbiased; this is known as a doubly robust estimator [27]. Further, TMLE allows one to use nonparametric algorithms which do not make modeling assumptions (e.g., linearity, no interaction etc.) that are common in standard regression models. In these analyses we used an ensemble (i.e., ‘stacking’) of prediction algorithms. These included standard regression models both with and without interactions, generalized additive models, mean outcome models and tree-based algorithms. These models were used in both the exposure and outcome models. All results are presented with 95% confidence intervals estimated via the delta method and were adjusted for the clustered nature of the trial data via previously published methods [28].

To assess possible dose–response relationship between hemoglobin level and adverse outcomes we included baseline hemoglobin as a continuous variable in mixed effects logistic regression models to estimate adjusted (for all above confounders) dose–response curves. We used restricted cubic splines with 3 degrees of freedom to allow for non-linearity in the hemoglobin/outcome relationship and plotted marginal risk curves for each outcome. Due to software limitations, TMLE is not available for continuous exposures.

We used E-values to assess the strength of an unmeasured confounder (such as dietary or nutritional intake) between baseline hemoglobin and adverse outcomes (on the risk ratio scale) that would be needed to explain away our results [29]. These were applied to all points estimates and lower bounds of the 95% confidence intervals when these values were > 1.0.

All perinatal outcomes were analyzed similarly to the above. All data analysis was conducted using R statistical software version 4.0.3 [30] TMLE was conducted using the *drtmle* package. Following guidance from the American Statistical Association and current practice in leading epidemiological journals, no null-hypothesis significance tests were performed [31].

## Results

**Fig. 1:**
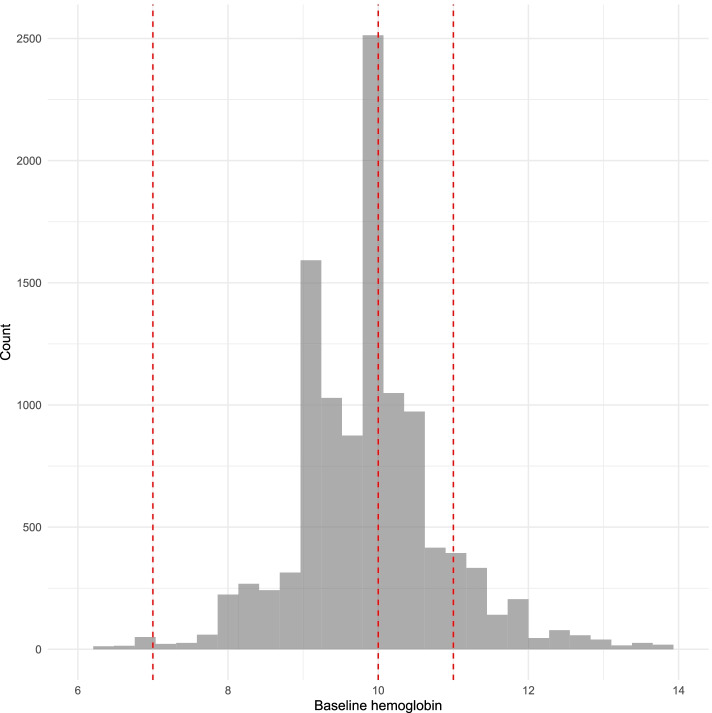
Distribution of baseline hemoglobin levels. Red dashed lines indicate cut-offs for (left to right) severe, moderate and mild anemia

A total of 14,783 pregnancies were enrolled in the trial. Of these, 13,017 (88.1%) had completed pregnancies within the trial period, and 11,370 delivered, while 1013 miscarried and 629 had a medically terminated pregnancy and 5 had unknown status. Among those who delivered, 11,085 had a baseline hemoglobin value; 10,066 (88.5%) were anemic at enrolment with the majority having moderate anemia (*n* = 6023, 59.8%) and only 68 (0.6%) having severe anemia. The distribution of hemoglobin levels was roughly bell-shaped, with observations mostly clustered between 8–12 g/dL (Fig. 1).

The majority of baseline characteristics were similar between those with varying levels of anemia. The only exceptions were parity and basic education (both maternal and husband), where anemic women were more likely to be parous and households with no anemia were more educated. Gestational age at delivery was similar in all groups (median of 39 weeks). There were decreasing rates of caesarean section with increasing anemia severity. Almost all (> 99%) delivered women were taking iron supplementation at time of delivery (Table 1). Women who miscarried or had medically terminated pregnancies had low rates of supplementation (25%, Table S2).

**Table 1:** Demographics and pregnancy characteristics by anemia level

	**None (*N* = 1304)**	**Mild (*N* = 3690)**	**Moderate (*N* = 6023)**	**Severe (*N* = 68)**
**Maternal age**	22.00 [20.00, 24.00]	22.00 [20.00, 25.00]	23.00 [20.00, 25.00]	23.00 [21.00, 26.00]
**Parous**	710 (54.5)	2306 (62.5)	3983 (66.1)	48 (70.6)
**Maternal basic education**	868 (66.6)	2197 (59.5)	3117 (51.8)	31 (45.6)
**Husband basic education**	884 (67.8)	2299 (62.3)	3362 (55.8)	37 (54.4)
**Multiple pregnancy**	12 (0.9)	35 (0.9)	49 (0.8)	1 (1.5)
**Body-mass-index**	19.95 [18.08, 22.20]	19.57 [17.80, 21.58]	19.11 [17.54, 21.00]	18.73 [17.34, 20.39]
**Iron supplementation **^**a**^	1290 (98.9)	3662 (99.2)	5969 (99.1)	68 (100.0)
**Religion**				
Hindu	1188 (91.1)	3335 (90.4)	5569 (92.5)	63 (92.6)
Muslim	109 (8.4)	336 (9.1)	423 (7.0)	5 (7.4)
Other	7 (0.5)	19 (0.5)	31 (0.5)	0 (0.0)
**Gestational age at enrolment**	11.14 [8.00, 15.86]	11.43 [8.43, 15.57]	11.57 [8.71, 16.00]	13.57 [9.57, 18.57]
**Gestational age at delivery**	39.00 [38.00, 40.00]	39.00 [38.00, 40.00]	39.00 [38.00, 40.00]	39.00 [36.75, 40.00]
**Mode of delivery**				
Vaginal	933 (71.5)	2760 (74.8)	4748 (78.8)	56 (82.4)
Vaginal (assisted)	12 (0.9)	40 (1.1)	51 (0.8)	1 (1.5)
Caesarian section	359 (27.5)	890 (24.1)	1224 (20.3)	11 (16.2)
**Trial arm**				
Intervention	705 (54.1)	2247 (60.1)	2814 (46.7)	32 (47.1)
Control	599 (45.9)	1443 (39.1)	3209 (53.3)	36 (52.9)
**Blood pressure measurement from POM**	644 (49.4)	2032 (55.1)	2592 (43.0)	27 (39.7)

^**a**^ Asked at time of delivery

Maternal morbidity occurred in roughly 5% of included pregnancies and rates were increased in all anemic groups. The three most common morbidities were blood transfusion, APH and sepsis; other morbidities were rare. There were nine maternal deaths (5 in mildly anemic and 4 in moderately anemic). Most women in the intervention arm had blood pressure measurements and this was consistent across levels of anemia severity. Hypertension was documented in around 10% of pregnancies and was highest in women without anemia. Postpartum hemorrhage was documented in less than 1% of pregnancies (Table 2).

**Table 2:** Maternal mortality and morbidity by anemia level

	**None (*N* = 1304)**	**Mild (*N* = 3690)**	**Moderate (*N* = 6023)**	**Severe (*N* = 68)**
Maternal mortality	0 (0%)	5 (0.1%)	4 (0.1%)	0 (0%)
Blood transfusion	32 (2.5%)	151 (4.1%)	269 (4.5%)	8 (11.8%)
Antepartum hemorrhage	13 (1.0%)	47 (1.3%)	39 (0.6%)	1 (1.5%)
Sepsis	14 (1.1%)	46 (1.2%)	65 (1.1%)	0 (0%)
Postpartum hemorrhage	8 (0.6%)	25 (0.7%)	42 (0.7%)	2 (2.9%)
Hypertension (POM) ^a^	80 (12.4%)	186 (9.2%)	223 (8.6%)	3 (11.1%)
Hypertension (expanded) ^a^	136 (21.0%)	318 (15.6%)	378 (14.5%)	3 (11.1%)
Pre-eclampsia ^a^	57 (8.8%)	130 (6.4%)	157 (6.0%)	3 (11.1%)

^**a**^ Only available in women in intervention arm with antepartum blood pressure measurements and not included in composite outcomes

The confounder adjusted association between early pregnancy anemia and adverse outcomes is provided in Table 3. For blood transfusion, there was increasing risk on both additive and multiplicative scales in all anemic groups compared to those with normal hemoglobin levels at baseline. There were no observed differences in either APH or sepsis, but confidence intervals were wide and consistent with both large increases and decreases in risk between groups. Results for postpartum hemorrhage were inconclusive in mild and moderately anemic women. Severely anemic women showed increased risk, but this estimate was based on only 2 events. Hypertension by both definitions was lower (compared to non-anemic) by between 20–25% in mild and moderately anemic women, comparisons of hypertension for severely anemic women defined by POM had wide confidence intervals and were uncertain. For the expanded definition, women with severe anemia had lower risk than those with no anemia. Risk of pre-eclampsia was reduced in mild and moderately anemic women by about 30% and was 37% higher in severely anemic women. For each of hypertension and pre-eclampsia, estimates for severely anemic women were based on only 3 events.

**Table 3:** Adjusted risk ratios and differences between women with no anemia and those with mild, moderate and severe

	**Unadjusted risk (%)**	**Risk difference % (95% CI) **^**a**^	**Risk ratio (95% CI) **^**a**^	**E-value point estimate **^**ab**^	**E-value lower bound **^**ab**^
**Blood transfusion**					
None	2.5	Reference	Reference	-	-
Mild	4.1	2.27 (1.29, 3.26)	2.16 (1.31, 3.56)	3.74	1.95
Moderate	4.5	2.68 (1.77, 3.58)	2.37 (1.56, 3.59)	4.17	2.50
Severe	11.8	9.20 (3.18, 15.23)	5.70 (3.00, 10.85)	10.9	5.45
**Antepartum hemorrhage**					
None	1.0	Reference	Reference	-	-
Mild	1.3	0.22 (-0.54, 0.97)	1.20 (0.63, 2.29)	-	-
Moderate	0.6	-0.39 (-1.14, 0.36)	0.65 (0.30, 1.39)	-	-
Severe	1.5	NA ^**abc**^	NA ^**abc**^	-	-
**Sepsis**					
None	1.1	Reference	Reference	-	-
Mild	1.2	0.01 (-0.69, 0.70)	1.01 (0.62, 1.65)	-	-
Moderate	1.1	-0.23 (-1.27, 0.82)	0.84 (0.36, 1.94)	-	-
Severe	0.0	NA ^**abc**^	NA ^**abc**^	-	-
**Postpartum hemorrhage**					
None	0.6	Reference	Reference	-	-
Mild	0.7	0.15 (-0.23, 0.54)	1.34 (0.60, 3.00)	-	-
Moderate	0.7	0.24 (-0.13, 0.61)	1.53 (0.69, 3.40)	-	-
Severe	2.9	1.72 (-0.89, 4.33)	4.82 (1.27, 18.3)	9.11	1.86
**Hypertension (POM) **^**abcd**^					
None	12.4	Reference	Reference	-	-
Mild	9.2	-3.11 (-4.15, -2.07)	0.75 (0.70, 0.80)	2.00	1.80
Moderate	8.6	-3.62 (-5.84, -1.39)	0.70 (0.58, 0.85)	2.21	1.63
Severe	11.1	0.01 (-7.12, 7.13)	1.00 (0.56, 1.79)	-	-
**Hypertension (expanded) **^**abcd**^					
None	21.0	Reference	Reference	-	-
Mild	15.6	-3.79 (-5.48, -2.1)	0.80 (0.73, 0.88)	1.80	1.53
Moderate	14.5	-4.35 (-6.24, -2.47)	0.77 (0.70, 0.85)		1.63
Severe	11.1	-7.72 (-13.77, -1.66)	0.60 (0.34, 1.05)	-	-
**Pre-eclampsia **^**abcd**^					
None	8.8	Reference	Reference	-	-
Mild	6.4	-2.13 (-5.14, 0.89)	0.75 (0.53, 1.07)	-	-
Moderate	6.0	-2.67 (-4.98, -0.35)	0.69 (0.55, 0.86)	2.25	1.60
Severe	11.1	3.20 (-1.23, 7.63)	1.37 (1.01, 1.88)	2.08	1.11
**Perinatal composite**					
None	19.9	Reference	Reference	-	-
Mild	17.5	-3.56 (-8.19, 1.07)	0.82 (0.65, 1.04)	-	-
Moderate	16.6	-2.94 (-7.38, 1.49)	0.85 (0.68, 1.07)	-	-
Severe	29.4	7.51 (-3.37, 18.39)	1.37 (0.89, 2.11)	-	-

^**a**^ Estimates are adjusted for trial arm, maternal age, nulliparity, body-mass-index, gestational age at enrolment, maternal basic education, husband basic education, multiple pregnancy, and religion

^**bc**^ Size of association between unmeasured confounder and outcome needed to move the point estimate or lower bound of confidence interval to 1.0 (i.e., compatible with a null effect). Only displayed for those estimates where the lower confidence bound is > 1

^**def**^ Could not be reliably estimated due to small sample size

^**ghij**^ Only available for women in the intervention arm with blood pressure measurement

Estimated E-values indicated that an unmeasured confounder beyond those included in our models would need to increase the risk of anemia and hypertension by a minimum of 60–90% on the risk ratio scale to explain away the observed associations (E-values for lower confidence bounds, Table 3).

**Fig. 2:**
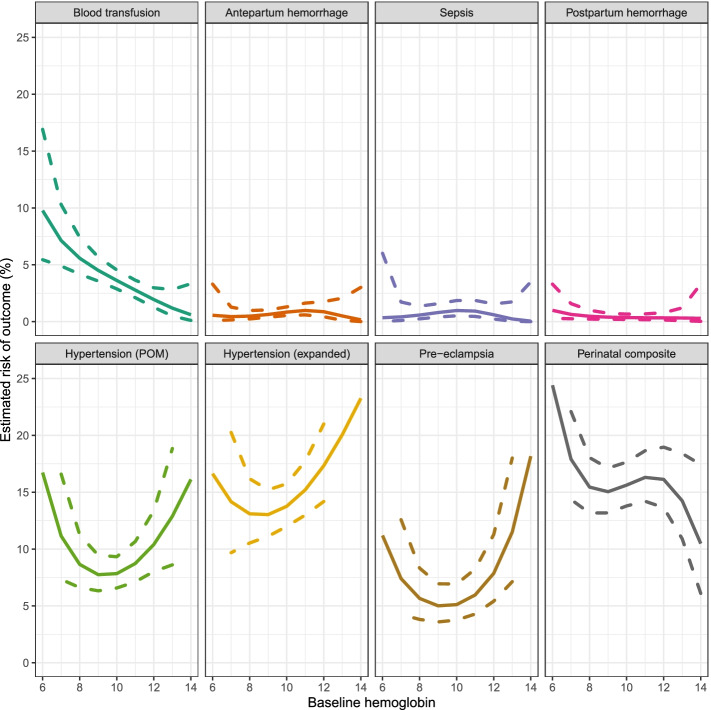
Estimated dose–response curves between hemoglobin level and maternal outcomes. Dashed lines represent 95% confidence intervals. Risk is adjusted for trial arm, maternal age, nulliparity, body-mass-index, gestational age at enrolment, maternal basic education, husband basic education, multiple pregnancy and religion

Dose–response relationships between hemoglobin and risk showed roughly linear increasing rates of the composite primary outcome with decreasing hemoglobin; however, confidence intervals at lower hemoglobin values were wide due to few measurements. Blood transfusion followed a similar pattern, while sepsis, APH and PPH estimated risks were relatively flat. There was a U-shaped relationship between hemoglobin level and hypertension (both definitions) and pre-eclampsia (Fig. 2). The inflection points for each of these outcomes was 10 g/dL. Estimated risks at each unit g/dL for each outcome are available in Table S3.

Adverse perinatal outcomes occurred in 20% of pregnancies with no anemia, 17% of pregnancies with mild or moderate anemia, and 29% of severely anemic pregnancies. Adjusted analyses for the relationship between anemia and perinatal death, stillbirth, early and late neonatal death and neonatal morbidity showed no consistent association as confidence intervals were too wide to draw meaningful inference (Table S4). Dose response curves for perinatal outcomes showed higher risk at very low hemoglobin values for all outcomes. For the composite perinatal outcome rates were flat for mild/moderate levels of anemia and declined for higher hemoglobin values; this was driven by neonatal morbidity as curves for perinatal death, stillbirth, and early and late neonatal deaths were mostly flat (Fig. 2 and S1).

## Discussion

### Summary of findings

Most women in the CLIP India trial had some form of anemia at the time of antenatal care booking, most commonly moderate and rarely, severe. Almost all women received iron supplementation by the time of delivery, regardless of baseline anemia, and in line with Indian guidelines for routine supplementation. In addition, just under 5% received blood transfusion, which was a greater number than those who experienced antepartum or postpartum bleeding, Compared with a normal hemoglobin level, each of mild, moderate, and severe anemia more frequently prompted receipt of maternal blood transfusion. Women with mild-moderate anemia had no excess of adverse pregnancy outcomes, including bleeding and sepsis, although women with severe anemia was associated with an increased risk of PPH. The findings were unlikely to be related to unmeasured confounders, particularly for severe anemia. However, we observed a ‘U-shaped’ relationship between anemia severity and pregnancy hypertension, and pre-eclampsia specifically, with women with mild- or moderate anemia having significantly lower rates.

Given the near 100% reported rate of iron supplementation in delivered women in this cohort, these data represent the estimated effect of early pregnancy anemia on outcomes in a population where public health efforts for supplementation in pregnancy have been successful.

### Comparison with the literature

The prevalence of anemia in pregnancy in India is amongst the highest in the world. Estimates have varied from a substantial proportion to most women in pregnancy, as in our study. In South India, Vindhya et al. estimated the prevalence of anemia in pregnant women to be 33.9%, with 48.4% of cases being mild, 49.5% moderate, and 2.1% severe [5]. Other studies have reported even higher rates of maternal anemia in India, up to 88%, similar to our study [2, 5] that demonstrate higher than previously published data from Belgaum and Bagalkote [32].

Many studies in India and elsewhere have examined the impact of maternal anemia on perinatal outcomes, such as stillbirth, low birth weight, small for gestational age, and preterm birth [8, 9, 33–36]. Far fewer studies have examined the impact of maternal anemia on maternal outcomes, even by systematic review of LMIC data [6, 15]. One recent review found a 190% increase in the odds of blood transfusion in anemic women [37], but they did not stratify by severity of anemia, as in our study.

The relationship between anemia and anemia severity on gestational hypertension and pre-eclampsia has been mixed. A large cohort study from India and Pakistan (110,033 anemic women) found a U-shaped relationship in Indian women (RRs = 1.89 95% CI = 1.12 to 3.18) for severe anemia, but ~ 1.0 for mild/moderate) but not in Pakistani women (RR = 1.18, 95% CI = 0.81 to 1.73 for severe anemia) [15]. Another smaller study case–control from Sudan (606 anemic women) found increases in pre-eclampsia severe anemia (OR = 3.6, 95% CI = 1.4 to 9.1), as well as possible increases with mild or moderate (OR = 1.60, 95% CI = 0.80 to 3.40) [38]. In contrast, Jung et al.’s review of nine studies found little difference in pre-eclampsia among anemic women (OR = 1.15 95% CI = 0.80 to 1.64); however, they did observe a U-shaped dose–response relationship [37] similar to ours and to the Indian cohort referenced above [15]. Mechanisms for the relationship between higher hemoglobin values and pregnancy hypertension may include poor nutrient supply to the placenta due to increased blood thixotropy, and production of reactive oxygen species, together with increased iron [39]. On the other hand, there has been suggestion that increased corticotropin released hormones associated with low hemoglobin may increase maternal and fetal stress, which can cause pregnancy hypertension [37, 40]. That said, published data on biological mechanisms are limited and further studies are needed to better understand this relationship.

It is clear that iron supplementation can increase hemoglobin values in pregnancy, but effectiveness is challenged by poor adherence, continuous access to iron tablets, use of doses lower than necessary, and oral (vs. parenteral) route of administration [41–43]. However, even when effective, the impact of anemia treatment on adverse maternal and perinatal outcomes remains uncertain. The most recent Cochrane review found that few studies have assessed the impact of iron supplementation on APH, blood transfusion, sepsis, or pre-eclampsia, and most did not focus on anemia in early pregnancy [18]. Our findings suggest that current management strategies for mild and moderate anemia are largely effective in reducing risk to that comparable to women with normal hemoglobin. However, more research is needed to understand the increased risk of hypertensive pregnancy in those with normal hemoglobin, and whether iron supplementation is related.

### Strengths and limitations

Our study has several strengths, including our large prospective cohort, standardized collection of blood pressure data using a device validated for use in pregnancy and pre-eclampsia, and detailed documentation of other pregnancy outcomes and possible confounders of the anemia-outcome relationship in a high-quality cluster randomized trial. Also, we report findings in the context of successful public health efforts to provide iron supplementation to all pregnant women in India, where malaria is not endemic.

Our study also has some limitations. First, our sample size of severely anemic women was small, and therefore despite the large effect sizes, caution should be taken in the extrapolation of findings related to this group, as they may be subject to sparse data bias. Conversely, just over 11% of women had normal hemoglobin, and few had a hemoglobin above 15 g/dL to fully evaluate the U-shaped relationship between anemia and pregnancy hypertension. Second, we lacked data on the exact timing of initiation of iron supplementation and associated adherence; stratification by such information may have provided further nuance to our findings. We found low rates of supplementation in miscarried or MTP pregnancies, which occur (by definition) before 20 weeks. Therefore, it is possible that most women began supplementation after this point. Third, we had no information on the reasons for transfusion, but in all groups, the number of women transfused exceeded those with clinical bleeding. Fourth, this is a secondary analysis of data from a clinical trial with different primary objectives, so we did not measure hemoglobin levels throughout pregnancy, or collect information about potential dietary mechanisms. Although we did adjust for vegetarian diet, there are risks of residual confounding due to unmeasured differences (such as diet and nutrition) between anemic and non-anemic women. Our E-values indicate that such variables would need to have strong effect above and beyond our adjustment to explain away the increased risk for severe anemia. For moderate and mild anemia's relationship with hypertension, E-values were more moderate (1.5–2.0), but still unlikely to explain away the U-shape. Finally, as all participants were prescribed iron supplementation, so we cannot comment on the relative benefits and risks of iron supplementation vs. no supplementation.

## Conclusion

In a cohort of women with habitual anemia, who reported routine iron supplementation and infrequent blood transfusion during pregnancy, mild-moderate anemia in early pregnancy (compared with normal Hb) is associated with similar pregnancy outcomes, but lower rates of pregnancy hypertension and pre-eclampsia. These findings suggest that new strategies should be developed for women with severe anemia in early pregnancy. Furthermore, routine iron supplementation for women with normal Hb in early pregnancy needs further exploration due to increased hypertension in a population with widely prevalent anemia.

## Supplementary Information

**Additional file 1.**

## Data Availability

The datasets used and/or analysed during the current study are not publicly available as the low outcomes rates of some outcomes may allow for identification and be a breach of confidentiality but are available from the corresponding author on reasonable request.
