# Supplementary material for: Anemia and adverse outcomes in pregnancy: subgroup analysis of the CLIP cluster-randomized trial in India
Source: BMC Pregnancy Childbirth. 2022 May 13;22:407. doi: 10.1186/s12884-022-04714-y (PMC9101819; doi:10.1186/s12884-022-04714-y)

Table S1: CLIP India working group

| **First and middle names** | **Last names** |
| --- | --- |
| Shashidhar G | Bannale |
| Keval S | Chougala |
| Vaibhav B | Dhamanekar |
| Anjali M | Joshi |
| Namdev A | Kamble |
| Gudadayya S | Kengapur |
| Uday S | Kudachi |
| Sphoorthi S | Mastiholi |
| Geetanjali I | Mungarwadi |
| Esperança | Sevene |
| Khátia | Munguambe |
| Charfudin | Sacoor |
| Eusébio | Macete |
| Helena | Boene |
| Felizarda | Amose |
| Orvalho | Augusto |
| Cassimo | Bique |
| Ana Ilda | Biz |
| Rogério | Chiaú |
| Silvestre | Cutana |
| Paulo | Filimone |
| Emília | Gonçálves |
| Marta | Macamo |
| Salésio | Macuacua |
| Sónia | Maculuve |
| Ernesto | Mandlate |
| Analisa | Matavele |
| Sibone | Mocumbi |
| Dulce | Mulungo |
| Zefanias | Nhamirre |
| Ariel | Nhancolo |
| Cláudio | Nkumbula |
| Vivalde | Nobela |
| Rosa | Pires |
| Corsino | Tchavana |
| Anifa | Vala |
| Faustino | Vilanculo |
| Rahat N | Qureshi |
| Sana | Sheikh |
| Zahra | Hoodbhoy |
| Imran | Ahmed |
| Amjad | Hussain |
| Javed | Memon |
| Farrukh | Raza |
| Olalekan O | Adetoro |
| John O | Sotunsa |
| Sharla K | Drebit |
| Chirag | Kariya |
| Mansun | Lui |
| Diane | Sawchuck |
| Ugochi V | Ukah |
| Mai-Lei | Woo Kinshella |
| Shafik | Dharamsi |
| Guy A | Dumont |
| Tabassum | Firoz |
| Ana Pilar | Betrán |
| Susheela M | Engelbrecht |
| Veronique | Filippi |
| William A | Grobman |
| Marian | Knight |
| Ana | Langer |
| Simon A | Lewin |
| Gwyneth | Lewis |
| Craig | Mitton |
| Nadine | Schuurman |
| James G | Thornton |
| France | Donnay |
| Romano N | Byaruhanga |
| Brian | Darlow |
| Eileen | Hutton |
| Mario | Merialdi |
| Lehana | Thabane |
| Kelly | Pickerill |
| Avinash | Kavi |
| Chandrashekhar | Karadiguddi |
| Sangamesh | Rakaraddi |
| Amit | Revankar |

Table S2: Iron supplementation rates between miscarried or medically terminated pregnancies (<20 weeks) and delivered pregnancies in various anemic groups.

|  | **Miscarried/MTP**  **(N = 1,642)** | **Delivered***  **(N = 11,370)** |
| --- | --- | --- |
| Iron supplementation at pregnancy end | 426 (26.0%) | 11273 (99.2%) |
| None | 67 (24.4%) | 1290 (98.9%) |
| Mild | 152 (25.6%) | 3662 (99.2%) |
| Moderate | 198 (26.1%) | 5969 (99.1%) |
| Severe | 3 (75%) | 68 (100%) |
| No iron supplementation at pregnancy end | 1215 (74.0%) | 96 (0.8%) |
| None | 208 (75.6%) | 14 (1.1%) |
| Mild | 441 (74.2%) | 27 (0.7%) |
| Moderate | 562 (73.9%) | 54 (0.9%) |
| Severe | 1 (25%) | 0 (0.0%) |

*Primary analysis cohort

Table S3: Adjusted risk (95% confidence interval) at various hemoglobin levels for hypertension and pre-eclampsia.

| **Baseline hemoglobin** | **Composite** | **Blood transfusion** | **Antepartum hemorrhage** | **Sepsis** | **Postpartum hemorrhage** | **Hypertension (POM)** | **Hypertension (expanded)** | **Pre-eclampsia** |
| --- | --- | --- | --- | --- | --- | --- | --- | --- |
| 4 | 29.04, (4.84, 76.69) | 24.54, (3.57, 74.05) | 4.29, (0.14, 58.59) | 0.57, (0, 98.38) | 4.29, (0.14, 58.59) | 50.4, (5.48, 94.68) | 20.87, (2.11, 76.33) | 22.96, (1.58, 84.7) |
| 5 | 15.68, (5.56, 37.02) | 14.72, (5.02, 36.06) | 1.87, (0.26, 12.06) | 0.37, (0, 41.88) | 1.87, (0.26, 12.06) | 28.55, (7.11, 67.61) | 15.49, (3.81, 45.92) | 12.94, (2.4, 47.35) |
| 6 | 10.08, (5.71, 17.17) | 9.76, (5.43, 16.94) | 1, (0.3, 3.3) | 0.35, (0.02, 5.13) | 1, (0.3, 3.3) | 16.73, (7.7, 32.6) | 12.35, (5.54, 25.27) | 7.89, (2.88, 19.87) |
| 7 | 7.69, (5.4, 10.83) | 7.13, (4.89, 10.29) | 0.64, (0.25, 1.58) | 0.42, (0.1, 1.67) | 0.64, (0.25, 1.58) | 11.15, (7.33, 16.61) | 10.59, (6.8, 16.12) | 5.37, (2.86, 9.83) |
| 8 | 6.63, (5.09, 8.6) | 5.58, (4.17, 7.42) | 0.47, (0.22, 1) | 0.58, (0.25, 1.36) | 0.47, (0.22, 1) | 8.65, (6.61, 11.25) | 9.72, (7.38, 12.71) | 4.11, (2.57, 6.5) |
| 9 | 6.08, (4.91, 7.51) | 4.51, (3.57, 5.69) | 0.4, (0.21, 0.74) | 0.8, (0.41, 1.58) | 0.4, (0.21, 0.74) | 7.75, (6.33, 9.46) | 9.5, (7.66, 11.73) | 3.54, (2.36, 5.29) |
| 10 | 5.55, (4.51, 6.8) | 3.62, (2.88, 4.55) | 0.36, (0.19, 0.67) | 0.98, (0.51, 1.86) | 0.36, (0.19, 0.67) | 7.84, (6.57, 9.33) | 9.79, (8.09, 11.8) | 3.43, (2.35, 4.99) |
| 11 | 4.69, (3.7, 5.94) | 2.78, (2.11, 3.65) | 0.34, (0.17, 0.69) | 0.93, (0.46, 1.86) | 0.34, (0.17, 0.69) | 8.73, (7.12, 10.66) | 10.52, (8.68, 12.71) | 3.71, (2.53, 5.4) |
| 12 | 3.44, (2.42, 4.86) | 1.94, (1.26, 2.98) | 0.33, (0.14, 0.79) | 0.6, (0.23, 1.55) | 0.33, (0.14, 0.79) | 10.4, (8.02, 13.39) | 11.69, (9.41, 14.42) | 4.44, (2.95, 6.64) |
| 13 | 2.02, (1, 4.05) | 1.19, (0.49, 2.84) | 0.32, (0.08, 1.22) | 0.24, (0.03, 1.62) | 0.32, (0.08, 1.22) | 12.89, (8.61, 18.84) | 13.27, (10.26, 17) | 5.86, (3.74, 9.06) |
| 14 | 0.88, (0.21, 3.59) | 0.61, (0.11, 3.38) | 0.29, (0.02, 3.31) | 0.05, (0, 2.8) | 0.29, (0.02, 3.31) | 16.13, (7.37, 31.73) | 15.24, (10.47, 21.66) | 8.39, (4.83, 14.2) |
| 15 | 0.27, (0.02, 3.38) | 0.25, (0.01, 5.23) | 0.24, (0, 16.19) | 0, (0, 8.57) | 0.24, (0, 16.19) | 19.87, (4.4, 57.2) | 17.52, (8.97, 31.43) | 12.87, (5.47, 27.38) |

Risk is adjusted for maternal age, nulliparity, body-mass-index, gestational age at enrolment, maternal basic education, husband basic education, multiple pregnancy and religion.

*Only available for women in intervention arm.

Table S4: Perinatal outcomes by hemoglobin status

|  | **Unadjusted risk**  **N (%)** | **Risk difference (95% CI)*** | **Risk ratio (95% CI)*** |
| --- | --- | --- | --- |
| **Perinatal composite** |  |  |  |
| None | 260 (19.9%) | 0.00 (ref) | 1.00 (ref) |
| Mild | 646 (17.5%) | -3.56 (-8.19, 1.07) | 0.82 (0.65, 1.04) |
| Moderate | 1001 (16.6%) | -2.94 (-7.38, 1.49) | 0.85 (0.68, 1.07) |
| Severe | 20 (29.4%) | 7.51 (-3.37, 18.39) | 1.37 (0.89, 2.11) |
| **Perinatal death** |  |  |  |
| None | 79 (6.1%) | 0.00 (ref) | 1.00 (ref) |
| Mild | 205 (5.6%) | -1.97 (-4.88, 0.94) | 0.72 (0.46, 1.13) |
| Moderate | 353 (5.9%) | -1.24 (-3.75, 1.27) | 0.83 (0.58, 1.18) |
| Severe | 7 (10.3%) | 1.79 (-1.74, 5.33) | 1.25 (0.81, 1.94) |
| **Stillbirth** |  |  |  |
| None | 44 (3.4%) | 0.00 (ref) | 1.00 (ref) |
| Mild | 103 (2.8%) | -1.56 (-4.17, 1.06) | 0.63 (0.32, 1.24) |
| Moderate | 187 (3.1%) | -1.06 (-3.4, 1.27) | 0.75 (0.42, 1.31) |
| Severe | 4 (5.9%) | 1.71 (-2.34, 5.75) | 1.41 (0.64, 3.11) |
| **Early neonatal death** |  |  |  |
| None | 28 (2.1%) | 0.00 (ref) | 1.00 (ref) |
| Mild | 87 (2.4%) | -0.06 (-1.10, 0.97) | 0.97 (0.62, 1.53) |
| Moderate | 130 (2.2%) | 0.02 (-0.77, 0.82) | 1.01 (0.71, 1.43) |
| Severe | 2 (2.9%) | 0.48 (-2.53, 3.49) | 1.21 (0.41, 3.60) |
| **Late neonatal death** |  |  |  |
| None | 8 (0.6%) | 0.00 (ref) | 1.00 (ref) |
| Mild | 16 (0.4%) | -0.21 (-0.74, 0.32) | 0.67 (0.27, 1.63) |
| Moderate | 37 (0.6%) | 0.00 (-0.60, 0.60) | 1.00 (0.38, 2.60) |
| Severe | 1 (1.5%) | 0.92 (-1.91, 3.76) | 2.46 (0.32, 18.93) |
| **Neonatal morbidity** |  |  |  |
| None | 216 (16.6%) | 0.00 (ref) | 1.00 (ref) |
| Mild | 541 (14.7%) | -1.81 (-6.46, 2.83) | 0.88 (0.66, 1.19) |
| Moderate | 807 (13.4%) | -1.65 (-5.78, 2.49) | 0.9 (0.69, 1.16) |
| Severe | 16 (23.5%) | 6.77 (-3.48, 17.01) | 1.43 (0.86, 2.39) |

*Estimates are adjusted for trial arm, maternal age, nulliparity, body-mass-index, gestational age at enrolment, maternal basic education, husband basic education, multiple pregnancy, and religion.

Figure S1: Directed acyclic graph for relationship between early pregnancy hemoglobin and adverse maternal outcomes. Grey indicates variables adjusted for, and white indicates unmeasured variables.

Figure S2: Dose response with perinatal outcomes by hemoglobin


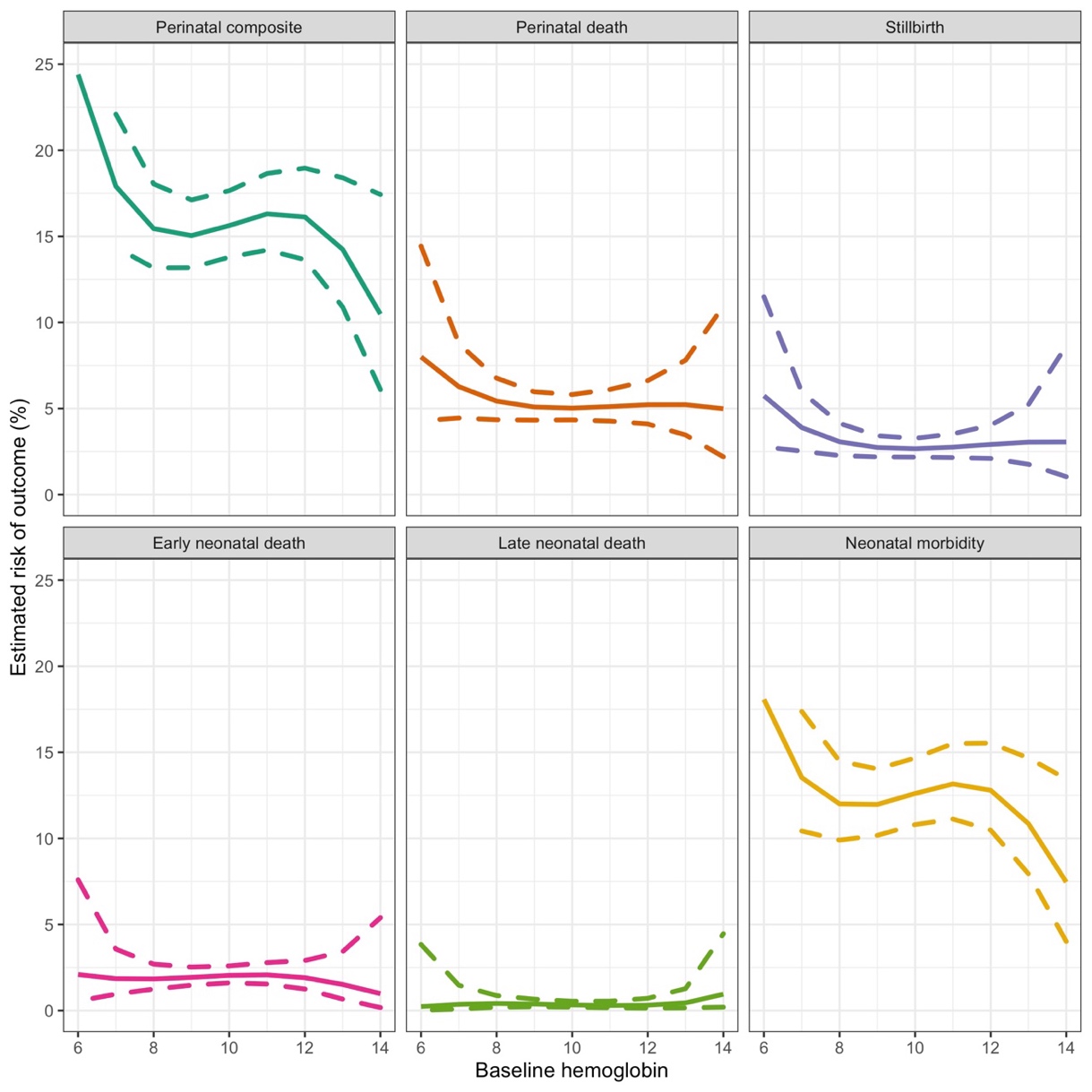

Supplement: Supplementary file 1 — Additional file 1. [file 12884_2022_4714_MOESM1_ESM.docx]
